# Associations of HLA-A, -B and -DRB1 Types with Oral Diseases in Swiss Adults

**DOI:** 10.1371/journal.pone.0103527

**Published:** 2014-07-29

**Authors:** Matti Mauramo, Adrian Markus Ramseier, Andreas Buser, Jean-Marie Tiercy, Roland Weiger, Tuomas Waltimo

**Affiliations:** 1 Department of Preventive Dentistry and Oral Microbiology, School of Dental Medicine, University of Basel, Basel, Switzerland; 2 Institute of Dentistry, University of Helsinki, Helsinki, Finland; 3 The Blood Transfusion Service SRC Basel, Basel, Switzerland; 4 National Reference Laboratory for Histocompatibility, Geneva University Hospital and University of Geneva, Geneva, Switzerland; 5 Clinic for Periodontology, Endodontology and Cariology, School of Dental Medicine, University of Basel, Basel, Switzerland; University of North Carolina at Chapel Hill, United States of America

## Abstract

Human leukocyte antigens (HLA) are crucial components of host defense against microbial challenge but the associations of HLA types with oral infectious diseases have not been studied in detail. This prospective cross-sectional study examined associations of HLA-A, -B and **-**DRB1 types with common oral diseases in a healthy Swiss adult population. 257 subjects (107 m, 150 f, mean age: 43.5 yr; range: 21–58 yr) with known HLA-A, -B and **-**DRB1 profiles and comprehensive medical records were included. A thorough anamnesis was followed by oral examinations including saliva flow measurements, the DMFT score for cariological status, complete periodontal status with plaque and bleeding indexes as well as assessment of mucosal alterations and temporomandibular dysfunction (TMD). Student’s t-test and Pearson chi-square test were utilized to compare the oral diseases between HLA positive and negative subjects. Bonferroni correction for multiple comparisons was used and P_Bonf_<0.05 was considered statistically significant. HLA types -B15 (P_Bonf_ = 0.002), -B51 (P_Bonf_ = 0.02) and -DRB1*12 (P_Bonf_ = 0.02) were associated with less periodontal disease manifestations. HLA-A32 had a positive association with TMD dysfunction (P_Bonf_ = 0.012). No other statistically significant associations were observed. In conclusion, HLA types may contribute to the development of oral diseases in generally healthy Caucasian adults.

## Introduction

Major histocompatibility complex (MHC) class I and II molecules, called human leukocyte antigens (HLA), have a pivotal role in immune response [1] [2] [3]. Pathogen-derived antigens are recognized by T-cells in the form of peptides that are bound to HLA molecules after antigen processing. HLA molecules vary extensively between individuals which may explain differences in inflammatory response towards microbial challenge [3] [4] [5] [6] [7]. However, the associations of HLA class I (HLA-A, -B and -C) and II antigens (HLA-DR, -DQ and -DP) with oral infectious diseases have not been studied extensively. Particularly, studies on possible HLA associations among generally healthy subjects are scarce.

Previously, associations have been documented between HLA antigens and periodontitis [8] [9] [10] [11]. A meta-analysis reported HLA class I types HLA-A9 and -B15 to indicate susceptibility to aggressive periodontitis, whereas HLA-A2 and -B5 were potential protective factors against aggressive periodontitis [12]. However, evidence on the associations of HLA types with dental caries is lacking. Contrasting results have been obtained on the associations of HLA class II antigens with salivary counts of cariogenic microbes, or with DMFT/S (decayed, missing or filled tooth/surface) score [11] [13] [14] [15] [16] [17] [18]. Among severely ill hemato-oncological patients prior to bone marrow transplantation we have observed an association of increased caries prevalence with the HLA types -A32, -B5, and -DR2, and a lower prevalence in HLA-B35 and -C4-positive individuals [10].

Hitherto, studies concerning the association of HLA types with oral diseases including caries and peridontitis have been based on a limited number of subjects or only on particular patient or population groups such as children or medically compromised sub-populations. Thus, these studies are not representative of generally healthy Caucasian adult populations. In addition, mucosal alteration, temporomandibular joint dysfunction (TMD) and saliva have not been subjected to epidemiological analyses in a sufficient scale. An objective of this preliminary study was to investigate associations of class I HLA-A and -B and class II HLA–DRB1 types with a broad range of oral diseases including periodontitis, caries, TMD and mucosal alteration as well as saliva flow in a population of healthy Swiss adults. Furhermore, this study was aimed to be an exploratory study without HLA-type-specific hypothesis.

## Subjects and Methods

### Ethics Statement

This prospective cross-sectional clinical study has been conducted according to the principles expressed in the Declaration of Helsinki, and has been approved by the Ethics Committee of Basel, Switzerland. Written informed consent was obtained from each subject.

### Subjects and HLA typing

Between 2009 and 2011, 2000 invitations were sent to consecutive subjects of the Swiss bone marrow donor register of Basel. 257 subjects volunteered to participate in the study. For the bone marrow donor register, the HLA types of the participants were already determined as follows: 1. HLA-A and -B types were determined by serology, i.e. by microlymphocytotoxicity using the Biotest AB120 trays (Biotest AG, Dreieich, Germany). 2. HLA-DRB1 types were obtained by PCR-sequence-specific oligonucleotide probe hybridization using commercial reagents (bioMérieux, Lyon, and One Lambda, Ingen, France). Because the majority of donors included in the study had been tested by DRB1 generic typing only, only low resolution level data were considered for the present analysis. Because of the very low antigen frequencies the DRB1*09, *10 and *16 allotypes were not computed in the final data analysis. Similarly the HLA-A (A25, A30, A33, A34, A66, A74) and HLA-B (B45, B46, B47, B48, B50, B52, B53, B54, B56, B58, B59, B61, B63, B67, B70, B73, B75, B76, B77, B78, B81, B82) antigens assigned with a low frequency or absent in the study group were not included in the analysis.

Anamnesis included oral hygiene (tooth brushing and flossing frequency) and dietary habits (frequency of eating sweets and/or confectionary), tobacco smoking (current, former, never), presence of chronic diseases and the use of any pharmaceutical products.

### Oral examinations

The clinical oral examinations of the patients were carried out by two calibrated clinicians (MM 204 subjects; AR 51 subjects). The recordings of the DMFT score for cariological status, mucosal alterations and temporomandibular dysfunction (TMD) followed the guidelines for oral health surveys of WHO [19]. In addition, a complete clinical periodontal status including probing pocket depth (PD) and clinical attachment level measurement (CAL) from six sites/tooth was performed with a pressure calibrated periodontal probe [20]. Oral hygiene was assessed by dental plaque present or not, according to O’Leary’s plaque control record [21] and bleeding on periodontal probing present or not, according to Ainamo and Bay [22]. Stimulated whole saliva flow rate (SWSFR) was determined as described by Sreebny and Vissink [23]. Briefly, subjects were asked to chew a commercially available, individually packed, neutral piece of paraffin wax (0.9 g/wax; Orion Diagnostica, Espoo, Finland) for one minute while swallowing all the saliva, followed by a five-minute period of chewing a new piece of wax and collecting the produced saliva in a graduated (mL) test tube (Sarstedt, Nümbrecht, Germany).

### Statistical methods

In all groups, HLA type frequencies were determined by counting the subjects being positive for a certain HLA allele. Mean ± standard deviation (SD) and 95% confidence intervals (CI) were calculated for metric variables. To ensure normal distribution only HLA types having >10 subjects were analyzed and the equality of variances was confirmed by the Levene’s test. Student’s t-test was performed for bivariate comparison between HLA positive and negative subjects. For dichotomous variables Pearson chi-square test was utilized. To determine statistical significance the Bonferroni correction for multiple comparisons was used ( = P_Bonf_), and P_Bonf_<0.05 was considered as statistically significant.

## Results

The subjects (107 m, 150 f, mean age: 43.5 yr; range: 21–58 yr) represented a generally healthy Swiss adult population with few self-reported medications (mean: 0.5, SD: 1.0, range: 0–10). 15% of the subjects were current, 27% former and 58% never smokers. Oral hygiene habits were generally good as 81% of the subjects reported brushing two or more times a day. However, relatively popular was also eating sweets and/or confectionary, as 42% reported consuming them more than 5 times a day and 51% 1–5 times/week (Detailed information of oral health related habits are shown in supportive information (Table S1)). The order of relative proportions of the most common HLA types (HLA-A01, -A02, -A03, -B07, -B35, -B44, -DRB1*01, -DRB1*04, -DRB1*07 and DRB1*13) was similar to a previous report in German-speaking Swiss population [24], suggesting our sample to be representative of this population. (For HLA frequencies, please see supportive information (Table S1); The original data available as supportive information (Table S2)).

### DMFT

No statistically significant associations of HLA-A, -B or -DRB1 alleles with DMFT score were found. HLA-A1 positive subjects were observed to have a tendency towards lower DMFT (mean DMFT: 12.9±5.5) compared to HLA-A1 negative subjects (mean DMFT: 14.9±7.0; P = 0.03, P_Bonf_ = n.s.,) However, after Bonferroni correction, the effect was not statistically significant (Table 1).

**Table 1:** DMFT, Stimulated whole saliva flow rates (SFR), TMJ symptoms and mucosal alterations of the subjects according to HLA-A, -B and -DRB1 types.

HLA type	N	DMFT					SFR, ml/min	TMJ clicking	TMJ symptoms	Mucosal alteration
		Mean	SD	95% CI		P*	mean (SD)	(%)	(%)	(%)
ALL	257	14.2	6.8				1.3 (0.7)	17.6	7.1	12.5
A01	69	12.9	5.5	−3.5	−0.2	0.03	1.3 (0.7)	17.4	8.7	11.6
A02	125	13.7	6.6	−2.7	0.7	0.23	1.3 (0.6)	12.8	7.2	12.8
A03	73	15.2	6.8	−0.5	3.2	0.16	1.3 (0.6)	19.2	5.5	12.3
A11	28	13.4	7.5	−4.0	2.0	0.51	1.4 (0.6)	14.3	3.6	10.7
A23	13	14.6	6.9	−3.8	4.6	0.84	1.5 (0.9)	7.7	15.4	7.7
A24	49	15.0	7.1	−1.4	3.0	0.47	1.4 (0.7)	12.2	6.1	12.2
A26	15	14.1	5.6	−3.3	3.1	0.94	1.7 (0.7)	26.7	6.7	0.0
A29	12	13.3	5.0	−4.3	2.2	0.50	1.0 (0.6)	0.0	0.0	33.3
A31	13	15.5	8.5	−3.9	6.5	0.60	1.3 (0.8)	30.8	15.4	30.8
A32	27	15.9	7.0	−1.0	4.8	0.20	1.2 (0.7)	40.7	3.7	14.8
A68	25	13.0	7.5	−4.6	1.8	0.37	1.3 (0.7)	20.0	4.0	20.0
B07	63	14.3	6.3	−1.8	1.9	0.95	1.3 (0.7)	14.3	4.8	12.7
B08	36	13.7	6.2	−2.9	1.6	0.58	1.3 (0.7)	19.4	8.3	13.9
B13	19	14.4	6.2	−2.9	3.3	0.89	1.4 (0.6)	10.5	10.5	10.5
B14	15	15.1	5.3	−2.1	4.0	0.52	1.4 (0.6)	33.3	13.3	20.0
B15	32	14.0	6.8	−2.8	2.4	0.86	1.5 (0.6)	21.9	9.4	6.3
B18	28	13.6	7.6	−3.7	2.4	0.66	1.2 (0.5)	10.7	3.6	10.7
B27	22	14.5	6.1	−2.6	3.1	0.86	1.4 (0.6)	22.7	0.0	22.7
B35	55	13.5	6.7	−3.0	1.1	0.37	1.3 (0.8)	16.4	1.8	12.7
B39	14	13.9	6.9	−4.4	3.7	0.87	1.3 (0.6)	7.1	7.1	0.0
B40	25	12.4	6.2	−4.7	0.7	0.14	1.2 (0.7)	20.0	12.0	12.0
B44	63	14.1	7.8	−2.3	2.0	0.89	1.4 (0.7)	19.0	7.9	19.0
B49	16	15.7	6.2	−1.9	4.9	0.35	1.4 (0.7)	25.0	6.3	12.5
B51	31	13.9	6.8	−3.0	2.2	0.77	1.4 (0.6)	9.7	12.9	12.9
B55	13	14.4	7.5	−4.5	4.7	0.94	1.4 (0.8)	23.1	15.4	15.4
B57	23	15.3	7.1	−2.0	4.3	0.47	1.3 (0.6)	13.0	8.7	8.7
DRB1*01	56	12.7	7.1	−4.0	0.2	0.07	1.3 (0.7)	17.9	7.1	10.7
DRB1*03	47	13.2	6.3	−3.4	0.7	0.21	1.3 (0.6)	21.3	6.4	19.1
DRB1*04	68	14.3	7.2	−1.9	2.1	0.92	1.4 (0.7)	14.7	11.8	14.7
DRB1*07	66	15.0	6.2	−0.8	2.8	0.29	1.3 (0.6)	12.1	6.1	15.2
DRB1*08	15	14.2	7.3	−4.2	4.1	0.98	1.1 (0.6)	13.3	0.0	6.7
DRB1*11	61	13.6	7.1	−2.9	1.2	0.42	1.2 (0.6)	19.7	4.9	9.8
DRB1*12	12	18.5	8.6	−1.0	10.0	0.10	1.2 (0.7)	41.7	8.3	25.0
DRB1*13	61	14.9	6.9	−1.1	2.8	0.40	1.3 (0.7)	16.4	6.6	18.0
DRB1*14	22	15.2	6.9	−2.1	4.2	0.51	1.3 (0.7)	18.2	4.5	9.1
DRB1*15	57	13.8	6.3	−2.5	1.4	0.55	1.3 (0.7)	17.5	5.3	12.3

*P value (two tailed t-test) between HLA genotype positive and negative subjects. No Bonferroni correction.

### Periodontal status

Less clinical attachment loss (CAL) was observed among subjects with HLA-B15 (mean number of sites with CAL ≥6 mm: 0.2±0.6; P_Bonf_<0.01) and HLA-DRB1*12 (mean number of sites with CAL ≥6 mm: 0.3±0.6; P_Bonf_ = 0.02) compared with the respective HLA type negative subjects (mean CAL ≥6 mm: 1.3±3.7; and 1.2±3.5, respectively). In addition, HLA-B40, -DRB1*01 and -DRB1*13 positive subjects had less clinical attachment loss of ≥4 mm and/or ≥6 mm compared with the respective HLA type negative subjects (Table 2). However, after the Bonferroni correction the differences were statistically not significant.

**Table 2:** Number of sites with periodontal attachment loss (CAL ≥4 mm and ≥6 mm) and gingival indexes of the subjects according to HLA-A, -B and -DRB1 types.

HLA type	N	CAL ≥4 mm					CAL ≥6 mm					PI**	PBI***	BOP****
		Mean	SD	95% CI		P*	Mean	SD	95% CI		P*	mean (SD)	mean (SD)	mean (SD)
ALL	257	6.4	9.9				1.1	3.5				0.2 (0.3)	0.2 (0.3)	0.2 (1.6)
A01	69	6.2	10.7	−3.2	2.6	0.85	1.6	5.3	−0.8	1.9	0.39	0.2 (0.2)	0.2 (0.3)	0.1 (1.0)
A02	125	5.9	9.0	−3.4	1.4	0.42	0.9	2.5	−1.3	0.3	0.24	0.3 (0.3)	0.2 (0.3)	0.1 (0.1)
A03	73	5.6	8.9	−3.7	1.4	0.36	0.9	2.4	−1.1	0.5	0.45	0.2 (0.2)	0.2 (0.2)	0.1 (0.1)
A11	28	7.5	13.9	−4.3	6.7	0.66	1.8	7.0	−2.0	3.5	0.60	0.2 (0.3)	0.1 (0.2)	1.0 (5.0)
A23	13	6.6	8.0	−4.8	5.2	0.94	1.3	3.4	−1.9	2.3	0.86	0.2 (0.2)	0.2 (0.3)	0.1 (0.1)
A24	49	6.2	10.2	−3.5	2.9	0.87	1.2	2.8	−0.9	1.0	0.96	0.3 (0.2)	0.2 (0.3)	0.6 (3.7)
A26	15	8.7	7.8	−2.0	6.9	0.26	1.2	1.7	−1.0	1.1	0.91	0.1 (0.2)	0.1 (0.3)	0.1 (0.1)
A29	12	13.0	14.5	−2.4	16.3	0.13	2.7	4.3	−1.2	4.4	0.23	0.3 (0.2)	0.2 (0.2)	0.1 (0.7)
A31	13	7.2	11.3	−6.2	7.7	0.82	0.6	1.5	−1.5	0.4	0.24	0.3 (0.2)	0.3 (0.2)	0.1 (0.1)
A32	27	7.7	8.9	−2.3	5.2	0.45	0.7	1.6	−1.3	0.3	0.22	0.2 (0.2)	0.3 (0.3)	0.2 (0.2)
A68	25	4.8	8.7	−5.6	2.0	0.35	0.7	1.5	−1.3	0.3	0.19	0.2 (0.2)	0.2 (0.2)	0.1 (0.1)
B07	63	5.9	7.1	−3.1	1.6	0.54	1.3	3.1	−0.7	1.1	0.66	0.2 (0.3)	0.2 (0.2)	0.1 (0.1)
B08	36	8.3	15.3	−3.1	7.5	0.40	2.6	7.4	−0.9	4.2	0.19	0.2 (0.3)	0.2 (0.3)	0.1 (0.1)
B13	19	7.2	10.5	−4.5	6.1	0.76	1.0	1.9	−1.2	0.9	0.76	0.3 (0.3)	0.2 (0.2)	0.1 (0.1)
B14	15	7.7	10.6	−4.6	7.3	0.63	1.3	2.1	−1.0	1.4	0.74	0.3 (0.4)	0.1 (0.2)	0.1 (0.1)
B15	32	3.4	6.3	−6.1	−0.9	0.01	0.2	0.6	−1.6	−0.5	0.00	0.3 (0.3)	0.2 (0.3)	0.1 (0.1)
B18	28	6.8	11.4	−4.2	5.0	0.85	1.9	5.1	−1.1	2.9	0.38	0.2 (0.2)	0.2 (0.3)	0.1 (0.1)
B27	22	7.1	8.4	−3.2	4.7	0.69	1.2	2.0	−0.9	1.1	0.86	0.3 (0.3)	0.2 (0.3)	0.1 (0.1)
B35	55	5.1	9.5	−4.6	1.2	0.24	1.2	5.3	−1.4	1.6	0.87	0.1 (0.2)	0.3 (0.4)	0.1 (0.1)
B39	14	8.3	11.1	−4.6	8.5	0.53	1.0	1.8	−1.3	1.0	0.78	0.3 (0.3)	0.2 (0.3)	0.6 (3.4)
B40	25	3.1	4.9	−6.1	−1.3	0.00	0.5	1.1	−1.4	−0.1	0.02	0.2 (0.2)	0.2 (0.3)	0.1 (0.1)
B44	63	8.1	11.9	−0.9	5.2	0.17	1.1	2.7	−0.9	0.8	0.84	0.2 (0.2)	0.2 (0.2)	0.1 (0.1)
B49	16	7.6	10.0	−4.3	6.7	0.65	1.2	3.1	−1.6	1.7	0.96	0.2 (0.3)	0.1 (0.1)	2.1 (7.2)
B51	31	4.1	8.9	−6.2	0.8	0.13	0.7	1.5	−1.3	0.2	0.12	0.3 (0.2)	0.2 (0.4)	0.1 (0.1)
B55	13	4.2	8.6	−7.7	3.0	0.36	0.7	1.7	−1.6	0.6	0.37	0.2 (0.2)	0.2 (0.2)	0.1 (0.1)
B57	23	8.0	9.9	−2.9	6.1	0.45	0.9	1.5	−1.1	0.6	0.53	0.2 (0.2)	0.1 (0.2)	0.1 (0.1)
DRB1*01	56	4.7	7.7	−4.8	0.3	0.08	0.5	1.3	−1.5	−0.2	0.01	0.3 (0.3)	0.2 (0.3)	0.1 (0.1)
DRB1*03	47	7.8	14.1	−2.6	6.0	0.44	2.5	6.9	−0.4	3.7	0.11	0.2 (0.2)	0.2 (0.2)	0.1 (0.2)
DRB1*04	68	8.0	10.0	−0.6	4.9	0.13	1.1	2.5	−0.9	0.7	0.85	0.3 (0.3)	0.3 (0.4)	0.2 (0.3)
DRB1*07	66	7.4	11.0	−1.7	4.3	0.39	1.3	2.7	−0.7	0.9	0.75	0.3 (0.3)	0.3 (0.4)	0.2 (0.2)
DRB1*08	15	4.9	6.7	−5.5	2.2	0.38	0.7	0.9	−1.2	0.2	0.13	0.2 (0.2)	0.1 (0.1)	0.1 (0.0)
DRB1*11	61	6.8	11.1	−2.7	3.5	0.79	1.4	3.7	−0.7	1.4	0.49	03 (0.3)	0.2 (0.4)	0.2 (0.4)
DRB1*12	12	5.3	6.2	−5.3	2.9	0.55	0.3	0.6	−1.5	−0.4	0.00	0.2 (0.2)	0.2 (0.2)	0.2 (0.2)
DRB1*13	61	4.8	6.3	−4.4	0.0	0.05	0.5	1.1	−1.4	−0.2	0.01	0.2 (0.2)	0.1 (0.1)	0.1 (0.1)
DRB1*14	22	4.8	12.7	−7.6	3.9	0.52	1.7	7.9	−2.8	4.2	0.71	0.2 (0.2)	0.1 (0.1)	0.1 (0.1)
DRB1*15	57	5.6	7.1	−3.4	1.3	0.39	1.1	2.5	−0.8	0.8	0.99	0.2 (0.2)	0.2 (0.3)	0.2 (0.3)

*P value (two tailed t-test) between HLA genotype positive and negative subjects. No Bonferroni correction.

**Plaque Index.

***Papillary bleeding Index.

****Bleeding on probing.

HLA-B51 was associated with shallower probing pocket depths (PD). Subjects with HLA-B51 had fewer periodontal pockets of ≥6 mm (mean number of pockets: 0.2±0.6; P_Bonf_ = 0.02) compared with HLA-B51-negative subjects (mean: 1.1±3.3). In addition, HLA-B62 and -DRB1*13 positive subjects had fewer deep periodontal pockets (≥4 mm and/or ≥6 mm) compared with the respective HLA type negative subjects. However, after the Bonferroni correction the differences were statistically not significant.

There were no differences in the indexes of periodontal health (papillary bleading index, bleeding on probing) between the HLA types (Table 2).

### Other oral diseases and conditions

HLA-A32 positive subjects had clicking in temporomandibular joint (TMJ) more frequently than HLA-A32 negative subjects (40.7% vs. 17.6%, P = 0.001, P_Bonf_ = 0.012). Clinical TMJ clicking, diagnosed according to WHO criteria [19], was also more prevalent in HLA-B14 positive subjects but this difference was not statistically significant (P = 0.042, P_Bonf_ = n.s.) (Table 1).

No statistically significant associations of HLA-A, -B or -DRB1 alleles with mucosal alterations were found. HLA-A29 increased the prevalence of mucosal alteration diagnosed according to the criteria of WHO, but this difference was not statistically significant (P = 0.012, P_Bonf_ = n.s.) (Table 1).

Stimulated whole saliva flow rates did not differ between the HLA types (Table 1).

## Discussion

This prospective clinical study examined associations of HLA-A, -B and -DRB1 types with oral diseases in a generally healthy Swiss adult population. The study focused particularly on the two most prevalent oral diseases, caries and peridontitis. In addition, temporomandibular dysfunctions, mucosal alterations as well as saliva flow rates were examined.

DMFT index was used to determine past and present need for cariological interventions. In previous studies, associations between HLA types and caries have been somewhat inconsistent. The earliest study observed HLA-DR4 to be associated with dental caries [8]. Later, HLA-DR4 has been shown to be associated also with early childhood caries [18]. A few studies have examined associations between HLA-DR4 and saliva levels of Streptococcus mutans with contrasting results [13] [14] [15] [17]. In our previous study, we observed a weak association between HLA-DR2 and an increased DMFT score among recipients of hematopoietic stem cell transplantation [10]. In addition, HLA class I antigens -A32, -B5 and -B35 were observed to be associated with altered DMFT [10]. In contrast, studies conducted among generally healthy African-American women (n = 186), young Japanese adults (n = 106) and a child population (n = 60) have failed to demonstrate associations of HLA class II antigens with DMFT/S score [11] [16] [17]. Our current results are in line with these later studies, since no statistically significant association of altered DMFT with any of the HLA-A, -B or -DRB1 types could be detected in the population of Caucasian adults. HLA-A1 may have a negative correlation with DMFT but this was, however, not confirmed after applying the Bonferroni correction. Altogether, caries is an example of a disease with multiple etiological factors including dietary and oral hygiene habits, mutans streptococci colonisation, strain virulence factors and oral saliva-associated defense mechanisms. Therefore, a strong association of any of the HLA types with caries would have been surprising.

Contrary to the etiology of caries, several host dependent but not acquired factors are considered to contribute to periodontal diseases. Studies have been conducted to identify HLA types that predispose patients to periodontal destruction, with varying results. A meta-analysis by Stein *et al*. found no associations between HLA types and chronic periodontitism [12]. However, in that study a positive association of HLA-B15 with aggressive periodontitis was observed. No aggressive periodontitis was seen in our study, but HLA-B15 positive subjects had less clinical attachment loss compared with HLA-B15 negative subjects. This partly controversial observation demonstrates better periodontal health of HLA-B15 positive subjects over a long period of time, as CAL measurement is not interfered by temporary and local irritation, hormonal factors or lack of dental treatment as readily as periodontal pocket depth measurements (PD). In the light of these studies, it seems that HLA-B15 positive subjects have a somewhat peculiar genetic response towards periodontal challenge, having mainly a high tolerance against microbes but being prone to aggressive forms of periodontitis. In the current study, also novel negative associations of HLA-B51 and -DRB1*12 with periodontal disease manifestations were observed. These observations warrant further studies. In addition, we can not exclude that the primary HLA association is with HLA-C alleles that are in preferential linkage disequilibrium with -B51, such as -C*14 and -C*15.

HLA types contribute to the development of rheumatic diseases and arthritis. For example, HLA-B27 is routinely used in diagnostics due to its close association with ankylosing spondylitis and spondyloarthropathy [25] [26]. Bacterial contamination has been suggested to be one possible mechanism for HLA contribution to temporomandibular dysfuction (TMD) [27]. Different bacteria including *Chlamydia trachomatis*, *Mycoplasma genitalium*, and other Mycoplasma species have been identified within temporomandibular joint synofial fluid and surrounding tissues [28] [29]. Two studies have been conducted to analyze the associations of HLA types with temporomandibular dysfunction (TMD). In patients with rheumatic diseases together with TMD and in patients with severe TMD several HLA-A, -B and -DR alleles have been found in increased frequency [27] [30]. In this study, subjective TMD symptoms, mouth opening and ‘clicking’ as a sign of TMD were recorded. Similarly to the previous studies, clicking in the temporomandibular joint was more prevalent in HLA-B14 positive subjects but this difference was not statistically significant. In addition, HLA-A32 positive subjects had clicking in the temporomandibular joint statistically significant more frequently than HLA-A32 negative subjects. No associations of HLA-A32 with TMD have been reported previously. However, due to the limited number of subjects with this symptom and respective ethical considerations, painless clicking was not further examined by radiological methods, but warrants further studies.

HLA molecules have also been associated with inflammatory diseases causing oral and gastrointestinal ulcers. HLA-B51 has been strongly associated with Bechet’s syndrome and recurrent oral ulcers [31] [32] [33]. In addition, HLA-DR8 has been associated with treatment resistant Crohn’s disease. In this study, any signs of oral mucosal lesions including ulcers, leukoplakia, lichen planus, absesses or other conditions were examined. However, no statistically significant associations of HLA-A, -B or -DR types with mucosal alterations could be detected.

In terms of subjects and scope, the current study is the most extensive that has been conducted in the field. In addition, the results can be considered representative of Swiss population, as the observed HLA frequencies resembled those previously reported in Swiss population [23]. However, the study has limitations due to both the diversity of HLA molecules in the investigated population and the confounding factors of the multicausal oral diseases. Thus, this study should be considered as a preliminary report on associations between HLA types and oral diseases, relying to clinical parameters globally recommended by the WHO and other international organisations for the screening of caries and periodontal diseases. However, when assessing the complex and delicate responses of host to infections, more sensitive measures might be useful. Consequently, this study warrants further analyzes of saliva and gingival crevicular fluid samples of the subjects to determinate the microbes and host immune responses in terms of matrix metalloproteinases and tissue inhibitory proteinases capable of predisposing to the common oral diseases. In addition, we aim to rescreen the subjects in 5 years using the present data as a basis for a longitudinal study design to define the disease progression affected by HLA type dependent host response.

In conclusion, the findings of this hitherto largest study are in line with most of the previous publications concerning caries and periodontitis, as HLA types had only limited associations with the prevalence of these diseases in a population of generally healthy adults. With regard to temporomandibular dysfunctions and mucosal lesions, the current study could not confirm the previously suggested associations of HLA types with the diseases. However, the present study found HLA types which may contribute to oral diseases among healthy adults. These include HLA-A1 in caries, HLA-B15, -B51 and -DRB1*12 in periodontitis and HLA-A32 in temporomandibular dysfunctions. The exact role and mechanisms of the HLA types in oral diseases still remain for exploration in future studies.

## Supporting Information

Table S1 Descriptives of the subjects according to HLA-A, -B and -DRB1 types.
(DOCX)

Table S2 Original data.
(XLSX)

## References

[1] Schwartz RH (1985) T-lymphocyte recognition of antigen in association with gene products of the major histocompatibility complex. Annu Rev Immunol 3: 237–261. 2415139 10.1146/annurev.iy.03.040185.001321

[2] Baines M, Ebringer A (1992) HLA and disease. Mol Aspects Med 13: 263–378. 1287396 10.1016/0098-2997(92)90003-i

[3] Klein J, Sato A (2000) The HLA system. First of two parts. N Engl J Med 343: 702–709. 10974135 10.1056/NEJM200009073431006

[4] Marsh SG, Albert ED, Bodmer WF, Bontrop RE, Dupont B, et al (2010) Nomenclature for factors of the HLA system, 2010. Tissue Antigens 75: 291–455. 20356336 10.1111/j.1399-0039.2010.01466.x PMC2848993

[5] Cooke GS, Hill AV (2001) Genetics of susceptibility to human infectious diseases. Nat. Rev. Genetics 2: 967–977. 11733749 10.1038/35103577

[6] Fischer GF, Mayr WR (2001) Molecular genetics of the HLA complex. Wien Klin Wochenschr 113: 814–824. 11732117

[7] Trowsdale J, Knight JC (2013) Major hitocompatibility complex genomics and human disease. Ann. Rev. Genomics Hum. Genet. 14: 301–323. 10.1146/annurev-genom-091212-153455 PMC4426292 23875801

[8] Lehner T, Lamb JR, Welsh KL, Batchelor RJ (1981) Association between HLA-DR antigens and helper cell activity in the control of dental caries. Nature 292: 770–772. 6973697 10.1038/292770a0

[9] Machulla HK, Stein J, Gautsch A, Langner J, Schaller HG, et al (2002) HLA-A, B, Cw, DRB1, DRB3/4/5, DQB1 in German patients suffering from rapidly progressive periodontitis (RPP) and adult periodontitis (AP). J Clin Periodontol 29: 573–579. 12296785 10.1034/j.1600-051x.2002.290614.x

[10] Dobr T, Passweg J, Weber, Tichelli A, Heim D, et al (2007) Oral health risks associated with HLA-types of patients undergoing hematopoietic stem cell transplantation. Eur J Haematol 78: 495–499. 17391311 10.1111/j.1600-0609.2007.00841.x

[11] Altun C, Guven G, Orkunoglu F, Cehreli ZC, Karaaslan A, et al (2008) Human leukocyte antigen class II alleles and dental caries in a child population. Pediatr Dent 30: 154–159. 18481581

[12] Stein JM, Machulla HK, Smeets R, Lampert F, Reichert S (2008) Human leukocyte antigen polymorphism in chronic and aggressive periodontitis among Caucasians: a meta-analysis. J Clin Periodontol 35: 183–192. 18190553 10.1111/j.1600-051X.2007.01189.x

[13] Wallengren ML, Ericson D, Forsberg B, Johnson U (1991) Human leukocyte antigens in relation to colonization by mutans streptococci in the oral cavity. Oral Microbiol Immunol 6: 292–294. 1820567 10.1111/j.1399-302x.1991.tb00495.x

[14] Wallengren ML, Johnson U, Ericson D (1997) HLA-DR4 and number of mutans streptococci in saliva among dental students and staff. Acta Odontol Scand 55: 296–298. 9370027 10.3109/00016359709114967

[15] Wallengren ML, Ericson D, Hamberg K, Johnson U (2001) HLA-DR4 and salivary immunoglobulin A reactions to oral streptococci. Oral Microbiol Immunol 16: 45–53. 11169139 10.1034/j.1399-302x.2001.160108.x

[16] Acton RT, Dasanayake AP, Harrison RA, Li Y, Roseman JM, et al (1999) Associations of MHC genes with levels of caries-inducing organisms and caries severity in African-American women. Hum Immunol 60: 984–999. 10566600 10.1016/s0198-8859(99)00088-9

[17] Ozawa Y, Chiba J, Sakamoto S (2001) HLA class II alleles and salivary numbers of mutans streptococci and lactobacilli among young adults in Japan. Oral Microbiol Immunol 16: 353–357. 11737658 10.1034/j.1399-302x.2001.160606.x

[18] Bagherian A, Nematollahi H, Afshari JT, Moheghi N (2008) Comparison of allele frequency for HLA-DR and HLA-DQ between patients with ECC and caries-free children. J Indian Soc Pedod Prev Dent 26: 18–21. 18408266 10.4103/0970-4388.40316

[19] WHO. Oral Health Surveys. Basic Methods. 4^th^ edition. Geneva: World Health Organization 1997.

[20] Kaner D, Christan C, Dietrich T, Bernimoulin JP, Kleber BM, et al (2007) Timing Affects the Clinical Outcome of Adjunctive Systemic Antibiotic Therapy for Generalized Aggressive Periodontitis. J Periodontol 78: 1201–1208. 17608574 10.1902/jop.2007.060437

[21] O‘Leary T, Drake R, Naylor S (1972) The plaque control record. J Periodontol 43: 38–39. 4500182 10.1902/jop.1972.43.1.38

[22] Ainamo J, Bay I (1975) Problems and proposals for recording gingivitis and plaque. Int Dent J 25: 229–235. 1058834

[23] Sreebny LM, Vissink A (2010) Dry mouth: The Malevolent Symptom: A Clinical Guide. Wiley-Bleckwell, Iowa, USA.

[24] Buhler S, Nunes JM, Nicoloso G, Tiercy JM, Sanchez-Mazas A (2012) The heterogeneous HLA genetic makeup of the Swiss population. PLoS One 7: e41400. 22848484 10.1371/journal.pone.0041400 PMC3405111

[25] Brewerton DA, Caffrey M, Hart FD, James DCO, Nicholls A, et al.. (1973) Ankylosing spondylitis and HLA-A27. Lancet 904–907. 10.1016/s0140-6736(73)91360-3 4123836

[26] Schlosstein L, Terasaki PI, Bluestone R, Pearson CM (1973) High association of an HL-A antigen, W27, with ankylosing spondylitis. N Engl J Med 288: 704–706. 4688372 10.1056/NEJM197304052881403

[27] Henry CH, Nikaein A, Wolford LM (2002) Analysis of human leukocyte antigens in patients with internal derangement of the temporomandibular joint. J Oral Maxillofac Surg 60: 778–783. 12089692 10.1053/joms.2002.33245

[28] Henry CH, Hudson AP, Gérard HC, Franco PF, Wolford LM (1999) Identification of Chlamydia trachomatis in the human temporomandibular joint. J Oral Maxillofac Surg 57: 683–688. 10368093 10.1016/s0278-2391(99)90432-9

[29] Kim SJ, Park YH, Hong SP, Cho BO, Park JW, et al (2003) The presence of bacteria in the synovial fluid of the temporomandibular joint and clinical significance: preliminary study. J Oral Maxillofac Surg 61: 1156–61. 14586850 10.1016/s0278-2391(03)00674-8

[30] Helenius LM, Hallikainen D, Helenius I, Meurman JH, Koskimies S, et al (2004) HLA-DRB1* alleles and temporomandibular joint erosion in patients with various rheumatic diseases. Scand J Rheumatol 33: 24–29. 15124939 10.1080/03009740310004603

[31] Poulter LW1, Lehner T (1989) Immunohistology of oral lesions from patients with recurrent oral ulcers and Behçet’s syndrome. Clin Exp Immunol 78: 189–195. 12412747 PMC1534655

[32] Sun A, Hsieh RP, Liu BY, Wang JT, Leu JS, et al (2000) Strong association of antiepithelial cell antibodies with HLA-DR3 or DR7 phenotype in patients with recurrent oral ulcers. J Formos Med Assoc 99: 290–294. 10870311

[33] Meador R, Ehrlich G, Von Feldt JM (2002) Behçet’s disease: immunopathologic and therapeutic aspects. Curr Rheumatol Rep 4: 47–54. 11798982 10.1007/s11926-002-0023-z

