# Supplementary material for: Associations of HLA-A, -B and -DRB1 Types with Oral Diseases in Swiss Adults
Source: PLoS One. 2014 Jul 29;9(7):e103527. doi: 10.1371/journal.pone.0103527 (PMC4114782; doi:10.1371/journal.pone.0103527)
Supplement: Table S1 — Descriptives of the subjects according to HLA-A, -B and -DRB1 types. (DOCX) [file pone.0103527.s001.docx]

Table S1. Descriptives of the subjects according to HLA-A, -B and -DRB1 types

| **HLA** | **n** | **Freq.** | **Age** | **Smoking (%)** | | | **Tooth Brushing (%)** | | | | **Flossing (%)** | | | **Sweets/confectionary (%)** | | |
| --- | --- | --- | --- | --- | --- | --- | --- | --- | --- | --- | --- | --- | --- | --- | --- | --- |
|  |  |  | mean (SD) | never | former | current | >2/day | 2/day | 1/day | <1/day | >5/wk | 1-5/wk | <1/wk | >5/wk | 1-5/wk | <1/wk |
| ALL | 257 | 1.00 | 43.5 (8.7) | 58.0 | 27.2 | 14.8 | 24.9 | 56.4 | 18.3 | 0.4 | 23.0 | 52.1 | 24.9 | 41.6 | 51.0 | 7.4 |
| A01 | 69 | 0.27 | 43.2 (8.8) | 58.0 | 27.5 | 14.5 | 18.8 | 63.8 | 15.9 | 1.4 | 24.6 | 52.2 | 23.2 | 34.8 | 55.1 | 10.1 |
| A02 | 125 | 0.49 | 42.9 (9.1) | 59.2 | 27.2 | 13.6 | 27.2 | 49.6 | 23.2 | 0.0 | 26.4 | 52.0 | 21.6 | 39.2 | 52.0 | 8.8 |
| A03 | 73 | 0.28 | 43.3 (9.0) | 60.3 | 24.7 | 15.1 | 23.3 | 56.2 | 20.5 | 0.0 | 21.9 | 53.4 | 24.7 | 47.9 | 46.6 | 5.5 |
| A11 | 28 | 0.11 | 42.7 (8.4) | 78.6 | 14.3 | 7.1 | 32.1 | 50.0 | 17.9 | 0.0 | 28.6 | 50.0 | 21.4 | 53.6 | 46.4 | 0.0 |
| A23 | 13 | 0.05 | 46.4 (5.3) | 76.9 | 15.4 | 7.7 | 46.2 | 38.5 | 15.4 | 0.0 | 0.0 | 76.9 | 23.1 | 61.5 | 38.5 | 0.0 |
| A24 | 49 | 0.19 | 43.9 (8.3) | 61.2 | 28.6 | 10.2 | 20.4 | 59.2 | 20.4 | 0.0 | 22.4 | 53.1 | 24.5 | 46.9 | 46.9 | 6.1 |
| A26 | 15 | 0.06 | 42.5 (9.3) | 66.7 | 26.7 | 6.7 | 33.3 | 60.0 | 6.7 | 0.0 | 26.7 | 40.0 | 33.3 | 46.7 | 46.7 | 6.7 |
| A29 | 12 | 0.05 | 44.6 (8.3) | 41.7 | 41.7 | 16.7 | 0.0 | 91.7 | 8.3 | 0.0 | 33.3 | 50.0 | 16.7 | 33.3 | 66.7 | 0.0 |
| A31 | 13 | 0.05 | 47.3 (8.7) | 53.8 | 38.5 | 7.7 | 23.1 | 76.9 | 0.0 | 0.0 | 30.8 | 46.2 | 23.1 | 46.2 | 53.8 | 0.0 |
| A32 | 27 | 0.11 | 40.8 (5.9) | 48.1 | 29.6 | 22.2 | 29.6 | 66.7 | 3.7 | 0.0 | 18.5 | 51.9 | 29.6 | 48.1 | 48.1 | 3.7 |
| A68 | 25 | 0.10 | 44.9 (7.0) | 52.0 | 28.0 | 20.0 | 36.0 | 44.0 | 16.0 | 4.0 | 20.0 | 40.0 | 40.0 | 40.0 | 56.0 | 4.0 |
| B07 | 63 | 0.25 | 43.0 (8.4) | 61.9 | 22.2 | 15.9 | 23.8 | 55.6 | 20.6 | 0.0 | 20.6 | 55.6 | 23.8 | 39.7 | 54.0 | 6.3 |
| B08 | 36 | 0.14 | 42.9 (9.2) | 63.9 | 27.8 | 8.3 | 25.0 | 63.9 | 11.1 | 0.0 | 25.0 | 61.1 | 13.9 | 55.6 | 41.7 | 2.8 |
| B13 | 19 | 0.07 | 44.2 (8.9) | 47.4 | 31.6 | 21.1 | 21.1 | 36.8 | 42.1 | 0.0 | 36.8 | 47.4 | 15.8 | 36.8 | 52.6 | 10.5 |
| B14 | 15 | 0.06 | 47.8 (6.3) | 53.3 | 20.0 | 26.7 | 20.0 | 60.0 | 20.0 | 0.0 | 20.0 | 46.7 | 33.3 | 46.7 | 53.3 | 0.0 |
| B15 | 32 | 0.12 | 43.3 (9.8) | 53.1 | 28.1 | 18.8 | 31.3 | 43.8 | 25.0 | 0.0 | 31.3 | 53.1 | 15.6 | 46.9 | 40.6 | 12.5 |
| B18 | 28 | 0.11 | 42.8 (7.2) | 50.0 | 39.3 | 10.7 | 21.4 | 50.0 | 28.6 | 0.0 | 17.9 | 50.0 | 32.1 | 39.3 | 53.6 | 7.1 |
| B27 | 22 | 0.09 | 42.6 (7.9) | 59.1 | 27.3 | 13.6 | 27.3 | 54.5 | 13.6 | 4.5 | 9.1 | 59.1 | 31.8 | 50.0 | 45.5 | 4.5 |
| B35 | 55 | 0.21 | 46.1 (6.9) | 56.4 | 25.5 | 18.2 | 25.5 | 65.5 | 9.1 | 0.0 | 20.0 | 54.5 | 25.5 | 47.3 | 43.6 | 9.1 |
| B39 | 14 | 0.05 | 43.2 (9.9) | 50.0 | 50.0 | 0.0 | 28.6 | 50.0 | 21.4 | 0.0 | 7.1 | 64.3 | 28.6 | 35.7 | 50.0 | 14.3 |
| B40 | 25 | 0.10 | 44.8 (6.6) | 64.0 | 24.0 | 12.0 | 24.0 | 60.0 | 16.0 | 0.0 | 40.0 | 32.0 | 28.0 | 48.0 | 44.0 | 8.0 |
| B44 | 63 | 0.25 | 42.0 (8.3) | 61.9 | 27.0 | 11.1 | 22.2 | 60.3 | 17.5 | 0.0 | 20.6 | 55.6 | 23.8 | 34.9 | 60.3 | 4.8 |
| B49 | 16 | 0.06 | 42.2 (10.9) | 62.5 | 25.0 | 12.5 | 25.0 | 62.5 | 12.5 | 0.0 | 31.3 | 50.0 | 18.8 | 43.8 | 43.8 | 12.5 |
| B51 | 31 | 0.12 | 42.8 (10.0) | 71.0 | 22.6 | 6.5 | 25.8 | 45.2 | 25.8 | 3.2 | 29.0 | 41.9 | 29.0 | 45.2 | 48.4 | 6.5 |
| B55 | 13 | 0.05 | 42.5 (5.8) | 76.9 | 0.0 | 23.1 | 30.8 | 46.2 | 23.1 | 0.0 | 7.7 | 53.8 | 38.5 | 15.4 | 84.6 | 0.0 |
| B57 | 23 | 0.09 | 43.6 (6.8) | 39.1 | 39.1 | 21.7 | 26.1 | 65.2 | 8.7 | 0.0 | 26.1 | 56.5 | 17.4 | 21.7 | 65.2 | 13.0 |
| DRB1*01 | 56 | 0.22 | 42.1 (8.1) | 71.4 | 16.1 | 12.5 | 19.6 | 64.3 | 16.1 | 0.0 | 21.4 | 48.2 | 30.4 | 44.6 | 50.0 | 5.4 |
| DRB1*03 | 47 | 0.18 | 43.7 (8.3) | 55.3 | 29.8 | 14.9 | 25.5 | 57.4 | 17.0 | 0.0 | 21.3 | 55.3 | 23.4 | 46.8 | 48.9 | 4.3 |
| DRB1*04 | 68 | 0.26 | 43.8 (8.2) | 55.9 | 30.9 | 13.2 | 29.4 | 51.5 | 17.6 | 1.5 | 26.5 | 42.6 | 30.9 | 42.6 | 47.1 | 10.3 |
| DRB1*07 | 66 | 0.26 | 45.6 (8.1) | 47.0 | 31.8 | 21.2 | 30.3 | 53.0 | 16.7 | 0.0 | 31.8 | 51.5 | 16.7 | 31.8 | 57.6 | 10.6 |
| DRB1*08 | 15 | 0.06 | 43.0 (9.7) | 66.7 | 20.0 | 13.3 | 6.7 | 80.0 | 13.3 | 0.0 | 26.7 | 46.7 | 26.7 | 46.7 | 53.3 | 0.0 |
| DRB1*11 | 61 | 0.24 | 42.2 (9.3) | 57.4 | 24.6 | 18.0 | 24.6 | 59.0 | 14.8 | 1.6 | 14.8 | 52.5 | 32.8 | 34.4 | 60.7 | 4.9 |
| DRB1*12 | 12 | 0.05 | 44.5 (9.5) | 66.7 | 25.0 | 8.3 | 33.3 | 66.7 | 0.0 | 0.0 | 25.0 | 58.3 | 16.7 | 41.7 | 50.0 | 8.3 |
| DRB1*13 | 61 | 0.24 | 44.4 (8.6) | 63.9 | 27.9 | 8.2 | 24.6 | 54.1 | 21.3 | 0.0 | 24.6 | 50.8 | 24.6 | 45.9 | 44.3 | 9.8 |
| DRB1*14 | 22 | 0.09 | 45.0 (8.3) | 54.5 | 31.8 | 13.6 | 36.4 | 45.5 | 18.2 | 0.0 | 0.0 | 77.3 | 22.7 | 31.8 | 54.5 | 13.6 |
| DRB1*15 | 57 | 0.22 | 41.9 (9.9) | 52.6 | 28.1 | 19.3 | 21.1 | 56.1 | 22.8 | 0.0 | 21.1 | 54.4 | 24.6 | 43.9 | 49.1 | 7.0 |
